# Physiological Behavior of the Aquatic Plant *Azolla* *sp.* in Response to Organic and Inorganic Fertilizers

**DOI:** 10.3390/plants9070924

**Published:** 2020-07-21

**Authors:** Ehab Azab, Abdel-fatah Salah Soror

**Affiliations:** 1 Biotechnology Department, Faculty of Science, Taif University, P. O. Box 888, Taif, 21974 Taif, Saudi Arabia; 2 Botany and Microbiology Department, Faculty of Science, Zagazig University, Zagazig, 44519 Sharkia, Egypt; dr.abdelfatahsoror@gmail.com

**Keywords:** *Azolla*, organic fertilizer, inorganic fertilizer, biochemical composition

## Abstract

The present investigation aims to evaluate the impacts of organic and inorganic fertilizers on the water parameters and physiological behaviors of an aquatic plant (*Azolla sp*.). The experiment used three groups: treatment with organic or inorganic fertilizer and a group with no fertilization as a control. *Azolla sp*. were grown in cement ponds that received different treatments. For water analysis, the obtained results clarified that fertilization resulted in no variation in the temperature or total hardness among different treatments. Organic fertilizer increased the dissolved phosphorus content, total hardness, and bicarbonate alkalinity, as well as the total phosphorus content, whereas inorganic treatment increased the pH, total ammonia content, and total nitrogen content. Regarding the biochemical composition of *Azolla* *sp*., the chlorophyll content showed no variation among different treatment groups, while organic matter showed high variation among different treatment groups. The highest values for ash and fat content were recorded in control ponds. The highest protein content was found in organic treatment ponds. The addition of fertilizers led to an increase in the tissue contents of N and P compared to the control. This increase was highest when *Azolla sp*. was fertilized with organic fertilizer. The atomic N:P ratio was low in tissues subjected to either treatment compared with the control. The doubling time of *Azolla sp*. was decreased by fertilization. It is concluded that different fertilizer systems have significant effect on physico-chemical and biological parameters of water. Fertilization positively affects *Azolla sp*. growth. Organic fertilizer was more efficient for the growth of *Azolla sp*., so it can be considered as a source of biofertilizer and green manure in areas where it spreads.

## 1. Introduction

To enhance the biological productivity of aquatic plants grown in ponds, both organic manure and inorganic chemical fertilizers can be used [1,2]. The application of fertilizers stimulates pond productivity through autotrophic pathways as well as heterotrophic pathways [3,4]. The major fertilizing elements are phosphorus and nitrogen, which are applied to fishponds in the form of inorganic fertilizer to stimulate algal growth to increase zooplankton production. Moreover, phosphate fertilizer is important for regulating the productivity of fishponds. It is considered to be the most critical single factor in the maintenance of pond fertility [5]. However, nitrogen is not often considered to be a limiting nutrient in pond productivity [6,7,8], partly because it is biologically fixed by nitrogen-fixing blue green algae and bacteria occurring in the pond system [9] or is abundant in the bottom sediment due to the accumulation of decomposing materials. It could be stated that inorganic fertilizers contain higher contents of nitrogen and phosphorus than organic fertilizers, leading to a higher water quality and dissolved oxygen content within pH-moderating water [10]. Potassium and calcium can also be used as fertilizer elements. It could be stated that inorganic fertilizers have certain advantages compared with organic ones. Hence, inorganic chemical fertilizers are widely available, require low application rates, dilute easily in water and have well-defined compositions and high nitrogen contents. 

Organic fertilizers play an important role in the conservation of soil, since they improve the organic matter level and increase long-term soil fertility, affecting plant growth [11,12,13]. The addition of organic fertilizer to water plays an important role in the enhancement of the chemical, physical, and biological properties of water [14,15]. According to the source of organic fertilizer, both the stability and quality of fertilizer are affected based on the raw material composition [16,17]. Since organic wastes can pollute the surrounding environment, its management will benefit soil as a source of organic fertilizer that benefits soil and water fertility, as well as keeping the environment clean. Organic fertilizers that are widely obtainable within the Sharkia region are chicken, cow, and duck wastes. Therefore, the use of organic fertilizers to improve both soil fertility and productivity is gaining importance [18].

*Azolla pinnata* (*Azolla sp*.) is a small aquatic fern grown in aquatic ecosystems in tropical and subtropical regions [19,20,21]. It prefers still water with little flow and clay sandy or fertile soils [22]. The genus *Azolla sp*. is usually included with the genus Salivinia in the family Saliviniaceae order Saliviniales, but it is separated into the family Azollaceae [23]. *Azolla sp*. fix atmospheric nitrogen in association with the cyanobacterium *Anabaena azollae* [24]. The combination of *Azolla sp*. and nitrogen-fixing algae leads to a high rate of productivity [25]. It can be used to replace part or all of the inorganic nitrogenous fertilizer required for rice production through *Azolla–Anabaena* symbiosis in which *Azolla sp*. fixes atmospheric nitrogen [26,27]. The high nutritional valve and protein content of *Azolla* sp. make it an important feed supplement for an assortment of animals [28]

It can be stated that fertilized fishponds are a suitable medium for *Azolla sp*. growth; however*, Azolla* sp. rapidly self-propagates and doubles its biomass in a few days (3–10 days) [29]. *Azolla* ferns are used for crop production in Asia and as a supplement to poultry diets due to their ability to fix 2–3 kg of nitrogen [24]. *Azolla sp*. has also attracted the interest of fish producers due to its high protein content [30]. 

Organic and inorganic pollutants that contaminate both soil and water can be accessed by plant roots causing contamination and requiring phytoremediation [31,32,33,34]. Sood et al. (2012) [35] illustrated the ability of the aquatic macrophytes *Azolla sp*. to phytoextract polluted contaminates as an environmentally friendly option for restoring polluted aquatic resources. [36] Liu et al. (2008) introduced *Azolla sp*. as an ecological life controller due to its high capacity to release oxygen and also as a carbon source of the blue-green algae *Anabaena spp* [37]. 

Fertilization increases chlorophyll, nutrient contents (N and P), biochemical composition, and growth rate of *Azolla* species. Adding fertilizers to *Azolla sp*. enhances its nutrition value [38]. Moreover, it can be used as a biological filter for purifying water as well as providing food and shelter to fish and invertebrates [39]. 

The aim of this study was to evaluate the influences of different fertilizers, like organic and inorganic fertilizers, on the physiological behavior of the aquatic plant *Azolla sp*. grown within ponds as well as the physico-chemical changes in water that received different kinds of fertilizer. 

## 2. Results 

### 2.1. Physico-Chemical Analysis of Water

**Figure 1:**
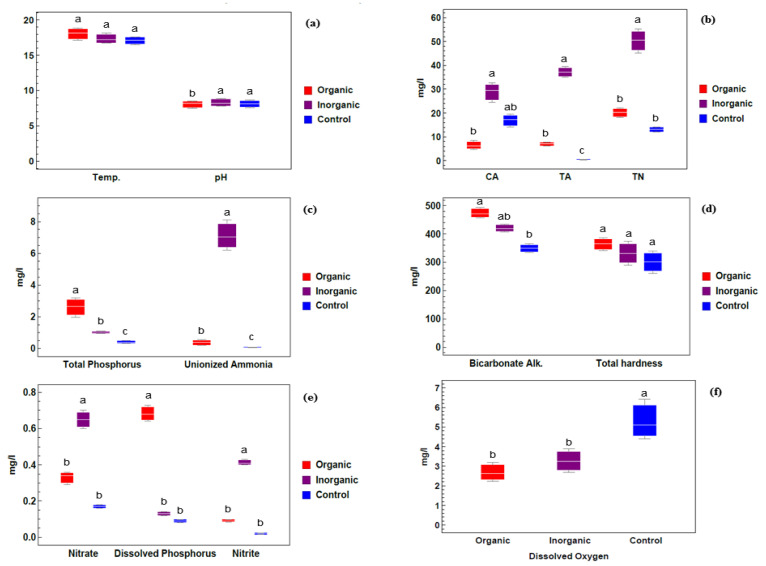
Physico-chemical parameters of water samples collected from different treatment groups: organic, inorganic and control. (**a**) Temperature, Hydrogen ion concentration; (**b**) CA (Carbonate Alkalinity), TA (Total Ammonia), TN (Total Nitrogen); (**c**) Total Phosphorus, Unionized Ammonia; (**d**) Bicarbonate alkalinity, Total hardness; (**e**) Nitrate, Dissolved Phosphorus, Nitrite; (**f**) Dissolved Oxygen. Different lowercase letters indicate significant differences between different treatments at *p* ≤ 0.05.

Water is the fundamental and most critical requirement for the growth of *Azolla sp*. The data shown in Figure 1a indicate that there were no differences in water temperature among the different treatment groups (organic and inorganic fertilizers and the control). Figure 1a shows that the hydrogen ion concentration was on the alkaline side and varied among different treatments. The maximum value was recorded with inorganic treatment (8.88), whereas the lowest value was observed with organic fertilizer treatment (8.30). 

As shown in Figure 1b, a higher carbonate alkalinity (CA) was found for the inorganic treatment group (32.83 mg/L) compared with the organic treatment (6.6 mg/L) and control (19.50 mg/L) groups and this was associated with a higher pH, while lower carbonate alkalinity was associated with a lower pH. On the other hand, the bicarbonate alkalinity (Bicarbonate Alk.) Figure 1d concentration was high in the organic treatment group (471.33 mg/L) and low in the control group (350.00 mg/L). 

Figure 1b also indicates that the total ammonia content (TA) had the highest value (39.4 mg/L) in the inorganic treatment group and the lowest value (0.55 mg/L) in the control group. Similarly, the unionized ammonia Figure 1c concentration was higher in the inorganic treatment group (7.02 mg/L) compared with the organic treatment (0.39 mg/L) and control (0.07 mg/L) groups. Highly significant variation was shown among different treatment groups. Moreover, as shown in Figure 1e, nitrite and nitrate concentrations were higher in the inorganic treatment group (0.41 and 0.65 mg/L, respectively) and lower in the control group (0.02 and 0.17 mg/L). In the same way, the total nitrogen content (TN) Figure 1b and unionized ammonia Figure 1c in the water supporting *Azolla sp*. recorded their highest values in the inorganic treatment group and their lowest values in control group. 

The total and dissolved phosphorus contents Figure 1c,e showed highly significant variations between the inorganic and organic treatment groups. The highest and lowest concentrations of dissolved phosphorus were 0.68 ± 0.08 and 0.09 ± 0.01 mg/L, and these were observed in the organic and control treatment groups, respectively. The higher total phosphorus content was measured in the organic treatment group (2.65 ± 0.43 mg/L), and regarding the total hardness Figure 1d, the lowest and highest concentrations were 302.66 ± 28.1 and 366.00 ± 15.6 mg/L in the control and organic treatment groups, respectively. From Figure 1f, we concluded that a higher value of dissolved oxygen was recorded in control ponds (5.15 ± 0.7 mg/L), while lower values were found in organic treatment ponds (2.63 ± 0.3 mg/L).

### 2.2. Azolla sp. Analysis 

#### 2.2.1. Chlorophyll Determination

**Figure 2:**
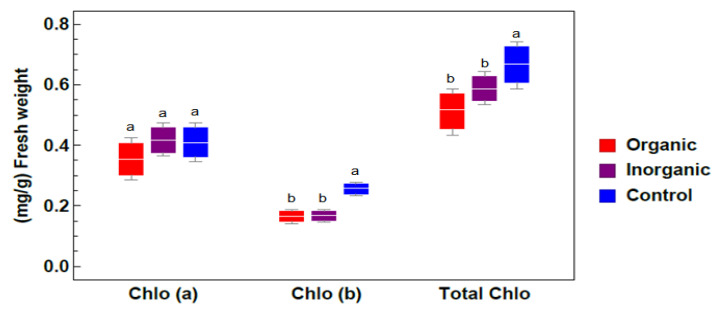
Impacts of different treatments on photosynthetic pigments in *Azolla sp*. plants. The photosynthetic pigments measured were chlorophyll a (Chlo a), chlorophyll b (Chlo b), and total chlorophyll (Total Chlo) (value (mg/g) ± the standard errors from three independent experiments under different treatments: organic, inorganic, and control). Different lowercase letters indicate significant differences between different treatments at *p* ≤ 0.05.

Regarding the chlorophyll concentration in *Azolla sp*. samples, the data given in Figure 2, which were used as a stress indicator, clearly indicate that chlorophyll a and b did not vary significantly among different treatment groups, and the maximum value (0.669 mg/g) was recorded in the control treatment group while the minimum value (0.519 mg/g) was found in the organic group. 

#### 2.2.2. Biochemical composition of Azolla sp. samples subjected to different treatments 

**Figure 3:**
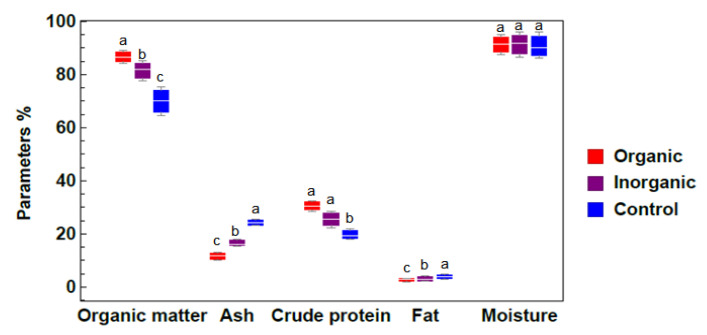
Biochemical composition of *Azolla sp*. samples exposed to different treatments (means ± standard errors from three independent experiments under different treatments: organic, inorganic and control). Different lowercase letters indicate significant differences between different treatments at *p* ≤ 0.05.

The results represented in Figure 3 show that organic matter varied significantly among different treatment groups, since the organic matter concentration was 86.53%, 82.03%, and 70.16% in the organic, inorganic, and control treatment groups, respectively. This can be seen by the percentage of protein content, since the highest protein content (30.25%) was found in the organic treatment group, followed by 25.57% and 19.25% in the inorganic and control group, respectively, whereas the fat and ash contents were high in the control treatment group (4.36% and 25.5%) compared with the inorganic and organic treatment groups (3.58%, 17.04%, and 3.06%, 12.07%, respectively; Figure 3). The highest protein content was found in the organic treatment group, followed by the inorganic and control groups. The fat content varied significantly (*p* < 0.05) among different treatment groups, and the highest values (4.36%) were recorded in control ponds. 

#### 2.2.3. Total Nitrogen and Total Phosphorus Contents

**Figure 4:**
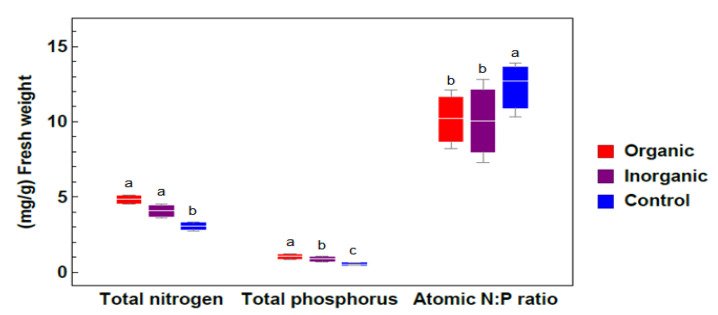
The variation of the total nitrogen (TN), total phosphorus (TP), and N: P ratio in tissues of *Azolla* sp. exposed to different treatments (means ± standard errors from three independent experiments under different treatments: organic, inorganic and control). Different lowercase letters indicate significant differences between different treatments at *p* ≤ 0.05.

Figure 4 shows that fertilization led to significant increases in both N and P in plant tissue content compared with the control. This increase was the highest when *Azolla sp*. was fertilized with chicken manure. The atomic N:P ratio in the tissues of both treatment groups was low (10.19 and 10.05) compared with the control (12.72), indicating a deficiency of N relative to P. Fertilization led to a decrease in the N:P ratio, where *Azolla sp*. absorbed more P than in control ponds. 

#### 2.2.4. Relative Growth Rate

**Figure 5:**
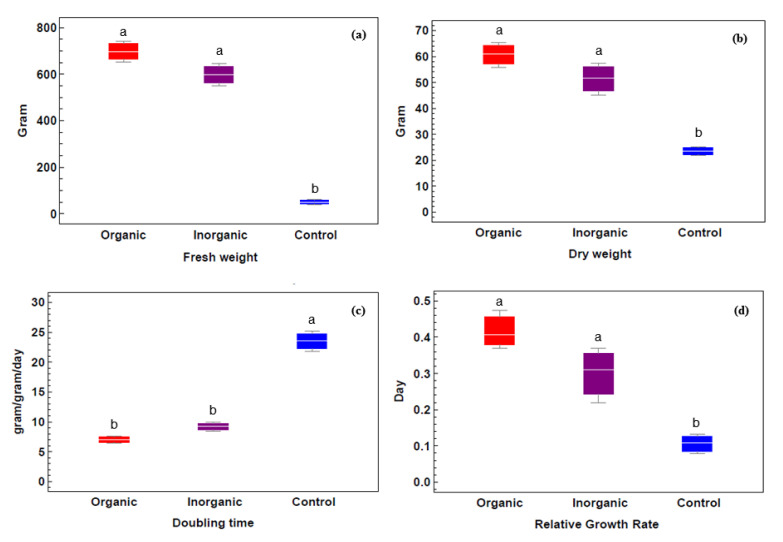
Relative growth rate and doubling time of *Azolla sp*. samples exposed to different treatments. (**a**) Fresh weight, (**b**) dry weight, (**c**) doubling time, and (**d**) relative growth rate of *Azolla sp*. samples under different treatments (means ± standard errors from three independent experiments under different treatments: organic, inorganic and control). Different lowercase letters indicate significant differences between different treatments at *p* ≤ 0.05.

In general, the two fertilized treatments showed increases in biomass compared with the control group, with the highest values shown for organic treatment for fresh weight (Figure 5a) and dry weight (Figure 5b). Figure 5c clarifies the decrease in the doubling time of *Azolla sp*. by using different fertilizers, ranging from an average of 7 d to 9.2 d for organic and inorganic treatments, respectively. The doubling time (D_t_) was 23.5 d in control ponds. The highest biomass values for fresh weight (698.26 g), dry weight (60.88 g), and relative growth rate (0.406 g/g/d) were observed in organic treatment ponds (Figure 5d). Moreover, there were no significant (*p* > 0.05) statistical differences in this area between the two fertilization treatments.

### 2.3. Correlation Coefficients

The correlations between each pair of variables are illustrated in Table 1. Positive correlations were identified between NH_4_ (ammonium) and NH_3_ (ammonia) (*r* = 0.996; *p* = 0.000), NO_2_ (nitrite) and NO_3_ (nitrate) (*r* = 0.982; *p* = 0.000), pH and CO_3_ (*r* = 0.978; *p* = 0.000), fresh and dry weights (*r* = 1.000; *p* = 0.000), and total nitrogen and protein (*r* = 1.000; *p* = 0.000). The relationship between relative growth rate (RGR) and doubling time (D_t_) was negative (*r* = −0.982; *p*= 0.000). On the other hand, there were no correlations between total phosphorus (TP) and total nitrogen (TN) (*r* = 0.266; *p* = 0.851), dissolved oxygen (DO) and NH_3_ (*r* = 0.365; *p* = 0.762), DO and TN (*r* = 0.451; *p* = 0.702), and DO and NO_2_ (*r* = 0.439; *p* = 0.711).

## 3. Discussion

According to the physico-chemical analysis of water, the hydrogen ion concentration was on the alkaline side in different treatments, which may be due to the negative effect of organic manure on the pH value of water, whereby the heterotrophic activities of aerobic bacteria reduce the pH through respiration through organic manure decomposition and serve as a continuous source of carbon dioxide, which leads to a decline in pH [40]. According to Utomo et al., 2019 [41], the pH in the studied ponds is suitable for the growth of *Azolla sp*., where productivity is maximized in water with a pH of 3.5–8. The bicarbonate alkalinity concentration was high in ponds treated with organic fertilizer due to the increased carbon dioxide production, resulting from organic fertilizer decomposition by bacteria. Carbon dioxide reacts with calcium and magnesium in water, forming calcium and magnesium bicarbonate in great volumes compared with inorganic treatments [41,42].

The values of different forms of nitrogen in the inorganic treatment group were increased compared with the organic and control groups. This may be a result of the high nitrogen content from the addition of urea, which dissolves in water and is rapidly converted to ammonia [43]. A higher total phosphorus content occurred in the organic treatment group, which may be due to organic fertilizer decomposition by bacteria which releases both forms of phosphorus [44]. Regarding the total hardness, the results are in accordance with Das et al. (2005), and Kamal et al. (2008) [45,46], who recorded increases in the total hardness and alkalinity with the addition of organic fertilizer. A lower value of dissolved oxygen was recorded with the organic treatment. This could be attributed to the lower dissolved oxygen content of the ponds with dense cover of floating plants than those with less cover of floating plants, which explains the increase in dissolved oxygen in the control compared with organic and inorganic treatment groups [47].

The maximum chlorophyll concentration in *Azolla sp*. samples occurred in the control group, while the minimum value was found in the organic treatment group. This may be due to the increased plant density (growth) in fertilized ponds which leads to dilution or distribution of chlorophyll pigment through the leaves [48,49]. Ren et al. (2017) [50] reported that with an increase in plant density, chlorophyll a and b contents significantly decreased, leading to a decreased photosynthetic rate during plant growth. These results are similar to those obtained for this study.

Organic matter showed highly significant variations among different treatments. Since the ash content was high in the control treatment compared with the inorganic and organic treatment groups, it seems that the effect of fertilization on the ash content was statistically significant. Similar responses were also found by Hazary (2015) [51], who reported that the ash content decreased with an increasing level of phosphorus. The highest protein content was found in the organic treatment group. It can be concluded that *Azolla sp*. plants can synthesize nitrogen compounds and transform them into protein form, even though the growing medium contains a low level of nitrogen. This may be due to the ability of *Azolla sp*. ferns to fix nitrogen in the presence of nitrogenase during symbiosis with *Anabaena* blue green algae as reported by [52]. In conclusion, different studies have illustrated that the environmental conditions affect the biochemical composition of *Azolla sp*. plants. Moreover, different species of *Azolla sp*. could have different compositions [53,54,55].

An N:P ratio lower than 8–16 indicates an N limitation, as illustrated in [56,57]. The lower N and P contents in the control group indicate the importance of nutrient availability for the growth of *Azolla sp*. Our results show that *Azolla sp*. is limited by N. The high contents of N and P in the tissues of *Azolla sp*. exposed to organic treatment indicate that this species is more capable of taking up these nutrients when exposed to organic fertilizer compared inorganic fertilizer [27,58]. The doubling time was very slow in control ponds (23.5 days), since [59] described that a D_t_ of 4–6 days is fast, 7–9 days is moderate, and more than nine days is slow.

In general, the two fertilized treatments were associated with an increase in biomass compared with the control group, with the highest values occurring with the organic treatment. There was no significant (*p* > 0.05) statistical difference between the two fertilized treatments. *Azolla sp*. growth increased following treatment with organic and inorganic fertilizers combined with high values of N and P content in the tissues. Similar results were obtained by [41] for organic and [60] inorganic fertilizer. Those authors mentioned that increased biomass of macroalgae tissues was observed after fertilization with N and P.

## 4. Materials and Methods

The experiment was conducted in nine concrete ponds. The volume of each pond was 12.5 m^3^ (5 m length; 2.5 m width; 1.0 m height). The ponds were located at the Aquaculture Research Laboratory, Abbassa, Abo-Hammad, Sharkia, Egypt. Firstly, ponds were drained and cleaned, and freshwater was added to a depth of 0.3 m. Thus, the volume of added water was 3.75 m^3^ = 3750 L. The experiment began in 1st November 2019 and continued for 30 days.

*Azolla sp*. plants were collected from irrigation canals and washed with 2% Clorox to kill attached organisms. The plants were then put inside the ponds to cover about 10% of the pond surface area*. Azolla sp*. was grown in three pond groups. The first group received 1.0 kg of poultry manure weekly as an organic fertilizer. The nutrient contents of poultry manure are shown in Table 2. The second group received inorganic fertilizer containing 20 mg/L urea (46.5% N) and 1.75 mg/L pure phosphorus (P) (45.0% P_2_O_5_), and finally, plants were grown in ponds without any fertilization as a control.

### 4.1. Sampling Points and Analysis

Three samples of *Azolla sp*. were collected weekly for each treatment (one sample from each pond) during one month to analyze the chlorophyll a and b contents, conduct a proximate chemical analysis (moisture, protein, fat, and ash %) in the dried *Azolla sp*., and measure the growth rate of *Azolla sp*. Water samples were also collected weekly to analyze the physico-chemical properties of water ponds throughout the investigation period.

#### Physico-Chemical Analysis of Water

The water temperature and dissolved oxygen were detected using an Oxygen-Thermometer apparatus (YSI model 58, Yellow Spring Instrument Co., Yellow Springs, Ohio, USA) [61]. The pH was measured using a glass electrode pH-meter [61]. The total alkalinity (mg/L) and total hardness (mg/L) were determined using the method described by [62]. The total ammonia concentration was measured [61]. The nitrite–nitrogen (NO_2_–N), nitrate–nitrogen (NO_3_–N), and dissolved phosphate (mg/L) concentrations were estimated as detailed in [63]. The total phosphorus and total nitrogen contents were determined as detailed in [64]. The dissolved phosphorus content was determined as described by [65].

### 4.2. Azolla sp. Analysis

#### 4.2.1. Chlorophyll Determination

Measurement of chlorophyll a and b was conducted as described by [66].

#### 4.2.2. Proximate Analysis

Samples of *Azolla sp*. plants were collected and analyzed to determine their moisture content % (by drying in an oven at 105 °C for 24 h), ash % (by ignition in a muffle furnace at 500 °C for 8 h), and fat content % (ether extract by the Soxhlet system using petroleum ether), and the Kjeldalh nitrogen content was estimated using the Kjeldalh method. Then, obtained values were multiplied by 6.25 to obtain the protein content values [67].

#### 4.2.3. Growth Rate

The total fresh biomass content in each pond was determined, and subsamples were taken for fresh weight and dry weight measurements. Fresh weight was determined by taking the plants out of the ponds and carefully blotting them dry on a paper towel before weighing them. The dry weight samples were washed in a sieve for 1 min while running demineralized water to remove adhered particles, and then the samples were put in paper bags in an oven for 48 h at 70 °C. The doubling time (D_t_) and relative growth rate (RGR) expressed as *g*/*g* per day were calculated using the formula reported by [68,69,70]:

D_t_ = 0.693 t/ln (B_f_/B_o_) (1)

where B_f_ is the final biomass; B_o_ is the initial biomass, and t is the growth period.

RGR = (lnB_2_ − lnB_1_)/(t_2_ − t_1_), (2)

where B_1_ and B_2_ represent the plant biomass at times t_1_ and t_2_ of the sampling period.

### 4.3. Statistical Analysis

By assuming that there is no precise difference in each concrete pond, samples collected from each treatment per month can be considered as dependent samples. Therefore, for the three independent treatments (control, organic, and inorganic), one way ANOVA and the Duncan multiple range test [71] were used to test whether differences among treatments and time were significant at *p* ≤ 0.05. Prior to ANOVA, data were tested for homogeneity using the Levene’s test. All the considered variables showed homogeneity. Specific post hoc comparisons were performed using LSD. Correlation coefficients and Pearson correlations (*r*) between the different parameters were computed. Correlations and all statistical analyses were conducted using SPSS for windows, version 22 (SPSS, Richmond).

## 5. Conclusions

In sum, the application of different fertilizers has a respectable effect on *Azolla sp*. productivity and water physico-chemical properties since fertilization increases the chlorophyll and nutrient contents (N and P), as well as improves the biochemical composition and growth rate of *Azolla sp*. These parameters were highest following organic fertilizer treatment compared with inorganic treatment. This fertilizer may be added to *Azolla sp*. grown in ponds to enhance its nutrition value, which enhances the protein content. Therefore, *Azolla pinnata* can be used as an alternative feed to animal food.

## Figures and Tables

**Table 1:** Pearson correlation coefficients (*r*) between each pair of different parameters.

Item	*r*	*p*
NH_4_^+^ & NH_3_	0.996	0.000
NO_2_^−^ & NO_3_^−^	0.982	0.000
pH & CO_3_^−2^	0.978	0.000
TP & TN	−0.266	0.851
DO & NH_3_	−0.365	0.762
DO & TN	−0.451	0.702
DO & NO_2_^−^	−0.439	0.711
RGR & D_t_	−0.982	0.000
Fresh & dry weight	1.000	0.000
TN & protein	1.000	0.000

Significant values at: *p* < 0.05, (*n* = 36). Data showed the correlation coefficients (*r*) and probability (*p*) between each pair of variables. NH_4_^+^; Ammonium; NH_3_: Ammonia; NO_2_^−^: Nitrite; NO_3_^−^: Nitrate; pH: Hydrogen ion concentration; CO_3_^−2^: Carbonate; TP: Total Phosphorus; TN: Total Nitrogen; DO: Dissolved Oxygen; RGR: relative growth rate; D_t_: doubling time.

**Table 2:** Nutrient contents of poultry manure.

**Organic Fertilizer Contents**	**Macronutrients**			**Micronutrients**			
	**N%**	**P%**	**K%**	**Fe (ppm)**	**Zn (ppm)**	**Cu (ppm)**	**Mn (ppm)**
	1.93	0.90	1.23	1150.6	185	33.3	222
